# Longitudinal Mood Monitoring and Classification of Self-Reported Mental Health Status Using Feels.ai: Feasibility Study

**DOI:** 10.7759/cureus.117257

**Published:** 2026-09-30

**Authors:** Peter Akpalu

**Affiliations:** 1 Internal Medicine, Independent Research, Chicago, USA

**Keywords:** behavioral monitoring, behavioral risk stratification, digital health, digital phenotyping, longitudinal monitoring, machine learning, mental health, mobile health, mood tracking

## Abstract

Purpose

This feasibility study evaluated the use of the Feels.ai (Chicago, Illinois) mobile app to collect longitudinal mood data and characterize emotional and behavioral patterns. A secondary objective was to explore whether app-derived behavioral features could distinguish participants with a self-reported mental health condition from those reporting none. Findings were intended to inform future validation studies focused on predictive modeling and clinical applicability.

Design

This retrospective observational study analyzed de-identified data from 61 college-aged adults who voluntarily enrolled in Feels.ai through social media outreach and recorded mood entries during routine app use. Mental health status was self-reported and was not independently verified through clinical assessment or standardized diagnostic instruments.

Methods

Participants recorded their mood by selecting from a series of emoji-based facial expressions that were internally mapped to a 0-10 distress scale for quantitative analysis. Numeric scores and descriptive labels were not displayed to participants. App-derived features included mood volatility, mean mood score, mood skewness, skipped days, and the day/night activity ratio. An exploratory random forest classifier was developed to distinguish participants reporting anxiety, depression, or both from those reporting none. Model training used repeated five-fold cross-validation with class upsampling. Missing predictor values were median-imputed within each cross-validation fold. Performance was evaluated using discrimination, classification, and calibration metrics. Logistic regression was evaluated as a conventional comparator using the same resampling and preprocessing approach.

Results

Across 61 participants, the average adherence rate was 69.7%, with a mean of 15.6 completed days. The random forest achieved an area under the curve (AUC) of 0.74 (95% CI, 0.62-0.86). At a screening-oriented cutoff of 0.30, sensitivity was 0.88 (95% CI, 0.73-0.97), specificity was 0.44 (95% CI, 0.25-0.65), and balanced accuracy was 0.66. The Brier score was 0.21, with a calibration intercept of 0.04 and a calibration slope of 0.59. Logistic regression achieved an AUC of 0.68, which was not significantly different from that of the random forest model (p = 0.27).

Conclusions

This study demonstrates the feasibility of using Feels.ai for longitudinal mood tracking and deriving behavioral features from routine app use. Exploratory modeling suggests that these features contain signals associated with self-reported mental health status. However, model performance and calibration should be interpreted cautiously given the small sample size, self-reported outcomes, missingness in some longitudinal features, and absence of external validation. Larger, more diverse prospective studies using validated clinical measures are needed to determine the reliability, generalizability, and potential clinical utility of these behavioral features.

## Introduction

Depression and anxiety are among the most prevalent mental health disorders worldwide, contributing significantly to disability, premature mortality, and the healthcare burden [1]. Mood and anxiety disorders frequently go undetected or untreated due to barriers to timely screening, stigma, limited access to care, and delays between symptom onset and clinical diagnosis [2,3]. Standard screening tools such as the Patient Health Questionnaire-9 (PHQ-9) [4] and the Generalized Anxiety Disorder-7 (GAD-7) [5] rely on retrospective self-report and intermittent administration, which may be susceptible to recall bias. Longitudinal mood tracking may complement these measures by providing additional information about day-to-day emotional variability [6-8].

Mobile health (mHealth) apps have emerged as accessible, scalable platforms for mental health monitoring and self-management, offering promising avenues to augment traditional screening and support early identification [6,7,9-14]. Digital mood-tracking platforms such as Moodscope have shown correlations with the PHQ-9 and GAD-7, supporting mobile-based mood monitoring outside clinical settings [6]. Similarly, the Immediate Mood Scaler (IMS) was validated as a brief mobile mood-tracking tool and demonstrated that mood ratings were strongly associated with PHQ-9 and GAD-7 scores [7]. Nonverbal mood assessment tools such as the Dynamic Visual Analogue Mood Scales (D-VAMS) have demonstrated validity and improved usability among individuals with low health literacy or cognitive burden, supporting the value of simplified affective measurement [15]. Furthermore, digital phenotyping research has shown that mood-relevant signals are reflected not only in subjective ratings but also in behavioral engagement patterns such as when, how often, and how consistently users interact with mobile tools [16].

Despite rapid growth in mHealth, most mental health apps lack empirical validation, clinical rigor, or transparency regarding their theoretical basis [17,18]. A recent systematic review found persistent methodological limitations in randomized controlled trials evaluating mental health apps, particularly in study design, replication, and reporting [19]. Additionally, many mental health apps are developed with limited empirical support, highlighting the need for evidence-based design, rigorous evaluation, and closer alignment with established clinical practice [20-22]. This persistent gap between commercial availability and scientific validation emphasizes the need for robust, data-driven tools that align with clinical standards.

Feels.ai (Chicago, Illinois) was developed to help address this need by enabling longitudinal mood monitoring through a simple nonverbal interface. Its visual response format was inspired by the Wong-Baker FACES Pain Rating Scale [23], which uses ordered facial expressions to support self-reported pain assessment. Feels.ai applies a similar visual-response concept to mood tracking with the aim of reducing cognitive burden and supporting frequent real-world use. By capturing mood entries in users’ natural environments, the app generates longitudinal self-reported data reflecting day-to-day emotional experiences. Feels.ai was developed to support scalable, real-world mood monitoring and exploratory feature extraction from routine user behavior.

The primary objective of this study was to evaluate the feasibility of Feels.ai for longitudinal mood monitoring and characterization of app-derived behavioral patterns. The secondary objective was to explore whether these behavioral features could distinguish participants with a self-reported mental health condition from those reporting none. The predictive analysis was exploratory and was not intended to identify or diagnose clinical mental health conditions.

About Feels.ai app

Feels.ai is a mobile mood-tracking application designed for brief, low-burden emotional check-ins using a proprietary emoji-based visual scale. Users record their mood by selecting from a series of facial expressions that are internally mapped to a 0-10 distress scale for quantitative analysis (Appendices). Numeric scores and descriptive labels were not displayed to users. The visual design was conceptually inspired by the simplicity of the Wong-Baker FACES Pain Rating Scale [23] but was independently developed for mood tracking. The app stores timestamped entries to support longitudinal analysis of mood and engagement patterns. Feels.ai was developed for general mood self-monitoring and was not designed as a clinical diagnostic or monitoring tool.

Every day, users are prompted to record a mood rating upon waking, with additional entries permitted throughout the day. If the morning check-in is missed, reminder nudges are issued every four hours to encourage mood logging. Users may also submit an optional selfie with each entry; however, selfie data were not analyzed in the current feasibility study.

## Materials and methods

Study design and participants

This study was a retrospective observational analysis of de-identified mood-tracking data collected through the Feels.ai mobile application. Participants were adults aged 18 years or older who voluntarily enrolled in Feels.ai through social media outreach (Instagram, Facebook, and Snapchat) and submitted at least one mood entry during the study period. Participants were neither recruited from a clinical population nor characterized as a clinical population in this study.

Data privacy and ethical considerations

All data were analyzed in aggregate, de-identified form, and no personally identifiable information was accessed. While users completed a baseline questionnaire that included demographic and self-reported mental health information, demographic variables were excluded from analysis to reduce identifiability. Feels.ai functioned as a self-reported mood-tracking application and was not used for clinical diagnosis, suicide-risk assessment, or treatment decision-making in this study.

Mental health group definitions

At baseline, participants self-reported mental health status by selecting one of four categories: anxiety, depression, both, or none of these. Participants reporting anxiety were categorized as “anxiety,” those reporting depression were categorized as “depression,” and those reporting both were categorized as “both.” Individuals reporting neither were categorized as “none.” However, these categories reflected self-reported mental health status and were not independently verified through clinical assessment or diagnostic interviews. Validated screening instruments such as the PHQ-9 [4] and GAD-7 [5] were not administered. Information regarding psychiatric comorbidities, treatment status, nutritional intake, physical activity, or suicidality was not collected. These factors were identified as priority measures for future studies. Accordingly, these categories should not be interpreted as clinically established diagnoses or measures of suicide risk.

Outcome measures

A set of quantitative indicators was derived from user-level mood logs to assess feasibility, engagement, adherence, and emotional variability throughout the tracking period. Engagement was characterized using multiple complementary measures, including total mood entries (the total number of mood ratings submitted), completed days (the number of unique days on which a participant submitted at least one mood entry), and total tracking days (the number of days between each user’s first and last recorded mood entry). Adherence rate was calculated as the ratio of completed days to total tracking days, with higher values indicating greater consistency in daily mood tracking. 

The mean mood score was calculated from emoji selections internally mapped to a 0-10 distress scale, serving as a proxy for overall emotional state. Mood volatility (variability) was quantified using the rolling standard deviation of daily mean mood scores over a three-day window to measure short-term fluctuations. Mood volatility therefore required at least three completed days for calculation. Mood skewness indicated whether a participant’s ratings tended to cluster toward the low-distress or high-distress end of the scale and required at least three mood observations for calculation. When insufficient observations were available, these features were treated as missing values.

Additional measures captured temporal and behavioral patterns of engagement. The day/night ratio compared daytime (6 AM-6 PM) and nighttime (6 PM-6 AM) mood entries, approximating circadian and sleep-related patterns. The longest streak corresponded to the maximum number of consecutive days with at least one logged mood entry, indicating periods of sustained engagement without interruption. Conversely, skipped days captured lapses in participation, defined as days without any logged mood entries within a participant’s tracking window. 

Data collection procedure

Data collection occurred between December 2023 and December 2024 using the Feels.ai mobile application. Participants rated their mood at least once a day using Feels.ai’s Visual Analog Scale featuring emoji facial expressions. Additionally, users had the option to log their mood multiple times throughout a given day. Mood entries were stored securely, anonymized, and analyzed in aggregate.

Behavioral features and exploratory modeling

Digital behavioral features were derived from participants’ longitudinal mood logs to characterize mood patterns and app engagement. Using all 61 participants’ aggregated feature data, an exploratory random forest classifier was developed to distinguish participants with a self-reported mental health condition (anxiety, depression, or both) from those reporting none. The model included mood skewness, day/night ratio, mood volatility, skipped days, and mean mood score as predictors. Participant identifiers and self-reported mental health group labels were excluded from the model predictors. Infinite predictor values were treated as missing. Missing predictor values were therefore median-imputed within cross-validation.

Model training used repeated five-fold cross-validation with 10 repeats and class balancing through upsampling. A fixed random seed (123) was used to support reproducibility. The random forest consisted of 1,000 trees and was optimized using the area under the receiver operating characteristic (ROC) curve (AUC). Out-of-fold predicted probabilities were aggregated at the participant level for performance evaluation.

Model performance was assessed using AUC, accuracy, sensitivity, specificity, and balanced accuracy. A 95% confidence interval for AUC was calculated using DeLong’s method. Exact binomial 95% confidence intervals were calculated for sensitivity and specificity. Probabilistic performance and calibration were further evaluated using the Brier score, calibration intercept, and calibration slope. A screening-oriented probability cutoff of 0.30 was used to calculate classification performance.

Logistic regression was evaluated as a conventional comparator using the same five predictors, preprocessing approach, and repeated cross-validation framework. Out-of-fold predictions were aggregated at the participant level and evaluated using the same performance and calibration metrics as the random forest model. AUCs from the two models were compared using a paired DeLong test.

Statistical analysis

Descriptive analyses, summary tables, and figure preparation were performed using MS Excel (Microsoft Corporation, Redmond, Washington). Statistical analyses and predictive modeling were conducted using R Version 4.5.0 (R Foundation for Statistical Computing, Vienna, Austria) within RStudio Version 2025.05.0+496 (Posit Software, PBC, Boston, Massachusetts).

## Results

Primary outcome: feasibility of data collection

The feasibility assessment examined engagement and adherence among participants grouped by self-reported mental health status. Key variables included averages of the number of mood entries, mood scores, adherence rate, total days tracked, completed days, and longest streaks of consecutive entries, as shown in Table 1.

**Table 1:** Feasibility metrics by average across mental health groups (n = 61).

Mental health	Number of mood entries	Mood score	Adherence rate	Total days	Completed days	Longest streak
Anxiety (n = 12)	20	4.0	80.4	24.6	17.8	7.8
Both (n = 13)	15.1	3.4	74.0	35.9	13.5	4.2
Depression (n* *= 9)	2.2	4.7	83.2	4.3	1.7	1.1
None (n = 27)	22.5	3.2	58.4	48.5	20.2	7.4

Across mental health groups, feasibility metrics suggested meaningful differences in engagement patterns and tracking consistency. Participants in the none group recorded the highest number of mood entries (M = 22.5), completed days (M = 20.2), and the longest observation windows (M = 48.5 total days), indicating the greatest sustained participation over time. The anxiety group also demonstrated strong engagement, averaging 20 mood entries and 17.8 completed days across 24.6 total days, along with the longest streak (7.8 days). Users reporting both showed moderate sustained engagement (15.1 mood entries, 13.5 completed days) over a longer tracking window (35.9 total days) but with shorter streaks (4.2 days), suggesting more intermittent participation.

The depression group demonstrated the lowest overall engagement (Figure 1), with the fewest mood entries (2.2) and completed days (1.7) across the shortest tracking window (4.3 total days). Although this group had the highest adherence rate (83.2%), this estimate likely reflects the limited observation period rather than sustained long-term tracking. The reduced participation among participants reporting depression highlights a potential challenge for sustained data collection. This supports further investigation of retention strategies such as tailored prompts, behavioral support, or low-burden engagement features.

**Figure 1:**
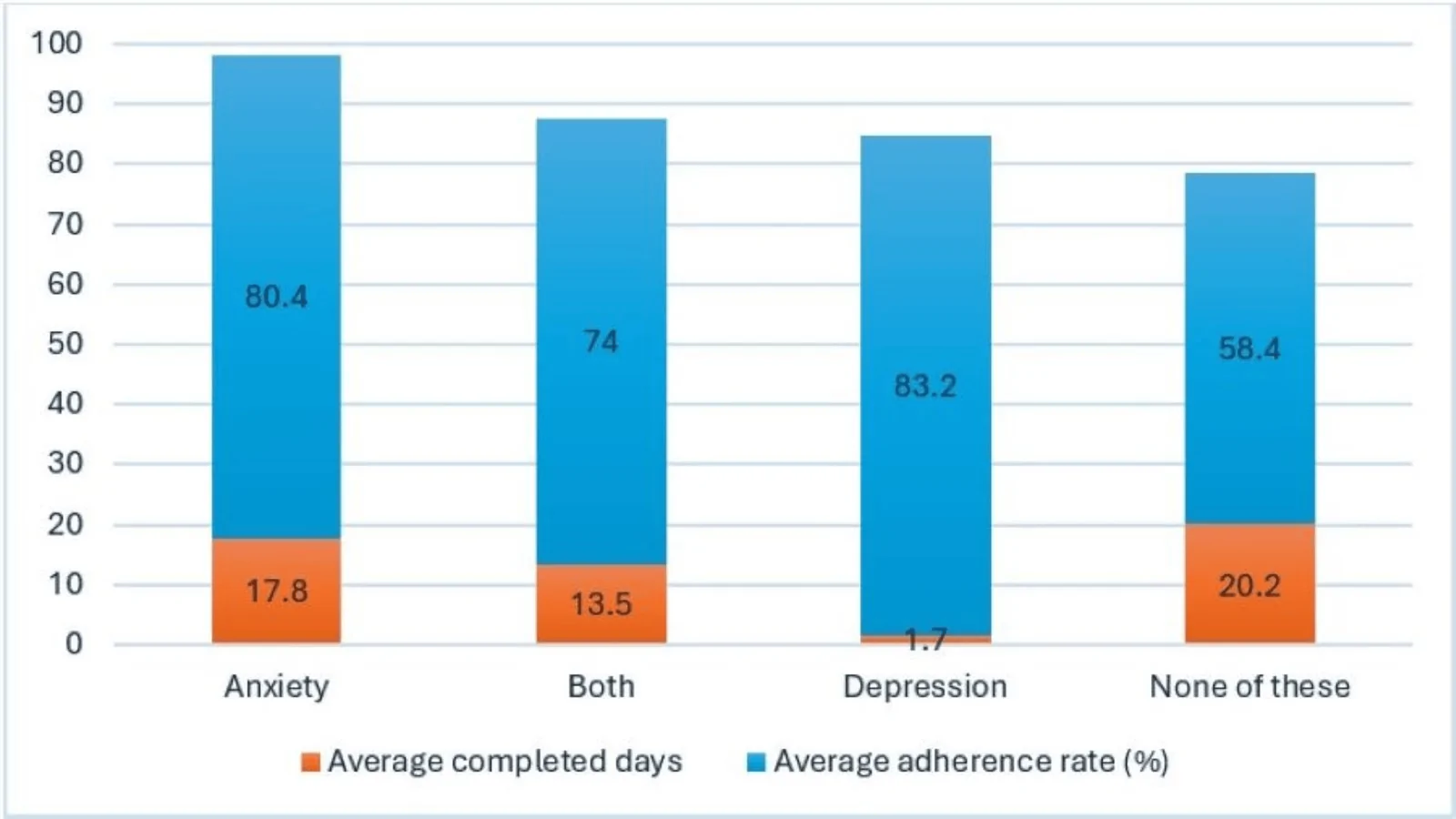
Engagement and adherence by mental health group.

Weekly engagement and mood trends

Weekday patterns among all users showed clear shifts in both engagement and mood (Figure 2). Users logged the most entries on Tuesday and stayed fairly consistent through Friday before dropping off over the weekend. Mood scores, with higher values reflecting more distress, followed a different rhythm. Distress was low at the start of the week, increased midweek on Wednesday and Friday, then dipped to its lowest point on Saturday before rising slightly on Sunday. These trends highlight how both mood and engagement varied across the week and suggest that weekends may warrant further investigation as a potential period for engagement-support strategies.

**Figure 2:**
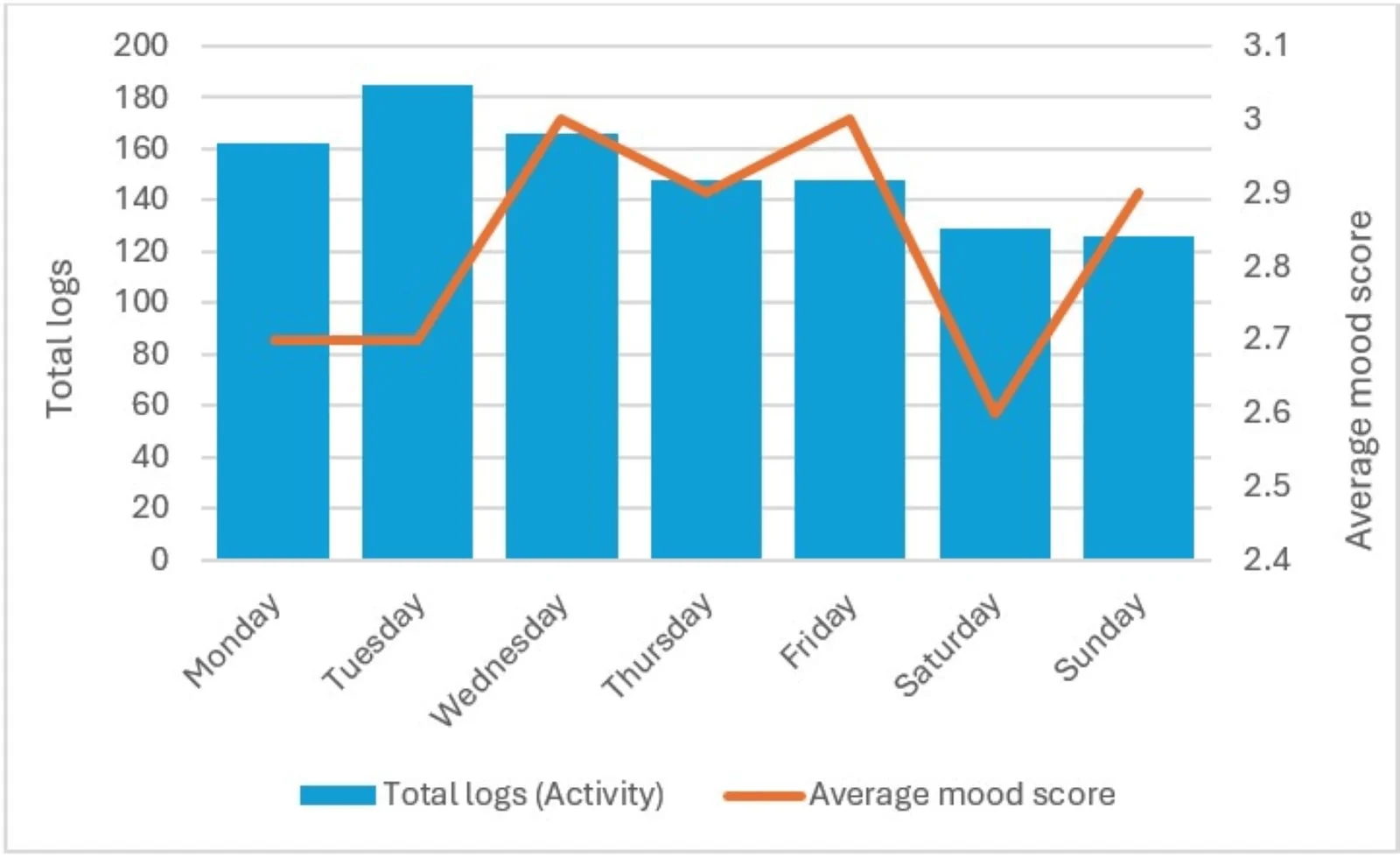
Weekly engagement and average mood scores (n = 61).

Secondary outcome: exploratory predictive modeling

To assess whether app-derived behavioral features could differentiate participants with self-reported mental health conditions from those reporting none, a random forest classifier was trained using feature-level data from all 61 participants. Features included mood skewness, day/night ratio, mood volatility, skipped-day patterns, and average mood score. As shown in Figure 3, the exploratory model achieved an out-of-fold area under the curve (AUC) of 0.74 (95% CI, 0.62-0.86). At a screening-oriented cutoff of 0.30, sensitivity was 0.88 (95% CI, 0.73-0.97), specificity was 0.44 (95% CI, 0.25-0.65), and balanced accuracy was 0.66. The Brier score was 0.21. Calibration analysis yielded an intercept of 0.04 (95% CI, -0.53 to 0.61) and a slope of 0.59 (95% CI, 0.22-0.96). Mood skewness was non-calculable for 22 of 61 participants (36.1%) and mood volatility for 27 of 61 (44.3%) because of insufficient observations. These values were treated as missing and median-imputed within cross-validation.

**Figure 3:**
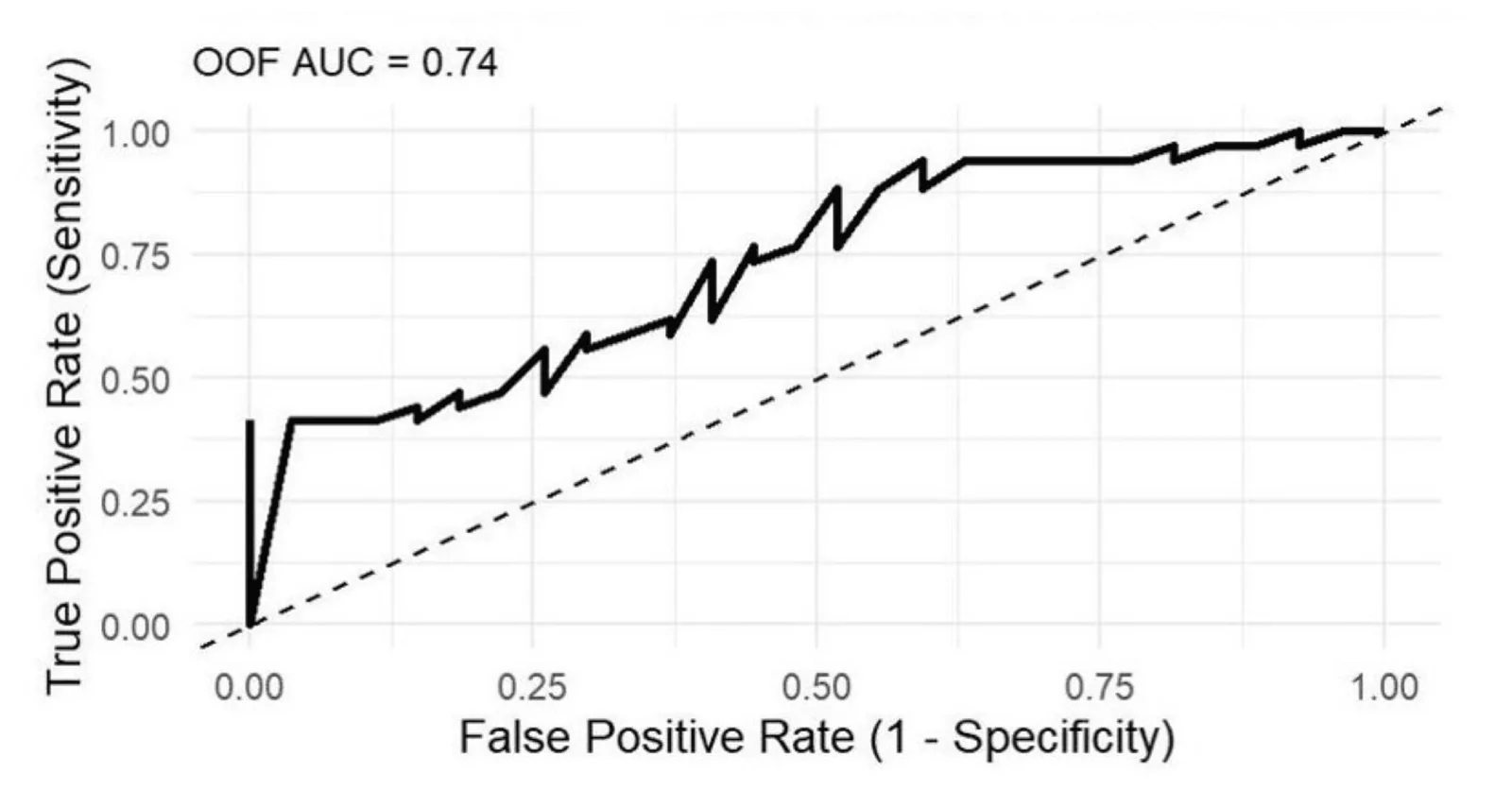
Receiver operating characteristic (ROC) curve for exploratory random forest model.

Analysis of feature importance (Table 2) showed that mood skewness and day/night ratio contributed most strongly to classification, followed by mood volatility and skipped-day patterns. Together, these findings suggest that app-derived behavioral features may contain signals for distinguishing self-reported mental health groups. However, model performance and feature importance should be interpreted cautiously given the small sample, limited calibration, and missingness in some longitudinal features.

**Table 2:** Random forest model feature importance.

Feature	Importance (%)
Mood skewness	100.0
Day/night ratio	55.2
Mood volatility	21.0
Skipped days	11.4
Mean mood score	0.0

As a conventional comparator, logistic regression using the same five predictors achieved an out-of-fold AUC of 0.68 (95% CI, 0.53-0.82). At the 0.30 cutoff, sensitivity was 0.85 (95% CI, 0.69-0.95), specificity was 0.30 (95% CI, 0.14-0.50), and balanced accuracy was 0.57. The Brier score was 0.24, with a calibration intercept of 0.21 (95% CI, -0.30 to 0.73) and a slope of 0.30 (95% CI, -0.12 to 0.72). Although the random forest demonstrated numerically higher discrimination, the difference in AUC between the models was not statistically significant (ΔAUC = 0.06, 95% CI, -0.05 to 0.17; p = 0.27).

## Discussion

Engagement and adherence patterns

A core challenge observed in this feasibility study was variability in engagement and adherence across mental health groups. While participants in the anxiety and none groups demonstrated relatively high engagement and completion rates, individuals in the depression group exhibited reduced activity. These findings are consistent with prior work by Torous et al. [24], which reported high dropout rates in mHealth interventions targeting depressive symptoms. More broadly, engagement remains a well-documented challenge in digital mental health interventions [12,13,22].

Consistent with Wu et al. [22], user engagement tends to decline over time in many mental health applications, particularly in the absence of personalization or behavioral reinforcement. Feels.ai currently relies on self-guided use without embedded engagement support, which may contribute to the reduced activity observed among users reporting depression.

Future iterations of Feels.ai may benefit from incorporating evidence-based engagement strategies, including personalized reminders, feedback loops, and low-burden interaction features. Prior research suggests that persuasive system design principles, including personalization, social support, and reward mechanisms, may improve user engagement and adherence in digital health interventions [22,25]. Additionally, simple interventions such as journaling, guided check-ins, and links to support resources have demonstrated utility in maintaining user interaction [9].

Within this exploratory model, temporal and engagement-related features contributed more strongly to classification than average mood scores. These results are consistent with emerging evidence that longitudinal behavioral features may provide additional information beyond static assessments, supporting their continued investigation in mobile sensing and digital phenotyping research [16,26,27].

Furthermore, skipped days were among the features contributing to model performance. This suggests that disengagement may not only represent a limitation of digital tools but may also contain potentially informative behavioral patterns. These findings raise the possibility that reduced engagement may provide behavioral information beyond poor adherence alone. As such, digital mental health tools may benefit from considering patterns of interaction alongside efforts to improve user retention.

Exploratory predictive modeling and digital phenotyping

Although preliminary, the exploratory machine learning analysis provides encouraging evidence that behavioral features derived from longitudinal app use may contribute to the classification of self-reported mental health status. The random forest model demonstrated modest discrimination, with an AUC of 0.74 (95% CI, 0.62-0.86). At the screening-oriented cutoff of 0.30, sensitivity was 0.88 (95% CI, 0.73-0.97), while specificity was 0.44 (95% CI, 0.25-0.65). The Brier score was 0.21. Calibration analysis yielded an intercept of 0.04 (95% CI, -0.53-0.61) and a slope of 0.59 (95% CI, 0.22-0.96). The calibration slope suggests overly extreme probability estimates, which may reflect model instability or overfitting in this small sample. These findings should also be interpreted in the context of substantial missingness in some longitudinal features.

Logistic regression was evaluated as a conventional comparator using the same behavioral features and cross-validation framework. The logistic regression model achieved an AUC of 0.68 (95% CI, 0.53-0.82), compared with 0.74 for the random forest model. Although the random forest demonstrated numerically better performance, the difference in AUC was not statistically significant (ΔAUC = 0.06, 95% CI, -0.05-0.17; p = 0.27). These findings suggest that the observed classification signal is preliminary and should not be interpreted as evidence of clinical validity.

These findings align with the growing field of digital phenotyping, which seeks to quantify behavioral and psychological states through longitudinal data collected from personal digital devices [16,26,27]. Prior studies have demonstrated that patterns in smartphone-derived behavioral data such as activity, sleep, and communication are associated with mental health status [28].

While this study was conducted in a non-clinical, self-reported sample, the observed behavioral patterns provide a basis for evaluating whether similar signals are present in clinically diagnosed populations. Future studies integrating longitudinal mood-tracking data with validated instruments such as the PHQ-9 [4] and GAD-7 [5] will be critical for determining whether these digital features offer additive value in real-world clinical screening and monitoring.

Future directions

Looking ahead, the integration of artificial intelligence with longitudinal mood-tracking data presents opportunities to enhance personalization and classification of self-reported mental health status. Emerging research has demonstrated the feasibility of using machine learning to detect depression from multimodal behavioral and communication data, including speech and language features [29]. These approaches suggest that multimodal data, such as behavioral patterns, self-reported mood, and passive signals, may improve predictive accuracy.

While such applications remain exploratory, future iterations of Feels.ai could incorporate additional data streams to complement longitudinal mood tracking. However, these developments should be approached with careful consideration of privacy, usability, and clinical validity. Future clinical applications should also incorporate appropriate safety resources and pathways for users experiencing suicidal thoughts or other acute mental health concerns. More broadly, these findings demonstrate the feasibility of deriving behavioral features from longitudinal mood-tracking data that warrant further investigation in relation to mental health status.

Limitations

Psychometric Validation

The emoji-based mood scale has not undergone formal psychometric validation. Its visual response format was inspired by the Wong-Baker FACES Pain Rating Scale [23]. However, the Wong-Baker scale was developed for pain assessment, and its established measurement properties cannot be assumed to extend to mood assessment. Individual interpretation of emoji expressions may also vary. Future prospective studies should evaluate the reliability and convergent validity of the Feels.ai scale against validated measures of mood and mental health symptoms.

Potential Effects of Repeated Mood Monitoring

The potential effects of repeated mood monitoring on participants’ emotional states were not assessed. Future studies should evaluate whether repeated self-monitoring influences mood or thought patterns.

Self-Report Bias 

Mood scores were collected through participant self-report and therefore remain subject to individual interpretation and reporting bias. Users may differ in how they perceive, label, or rate their emotional experiences. Responses may be influenced by factors such as social desirability, mood awareness, or personal interpretation of the scale. Although real-time mood tracking may reduce recall bias compared with retrospective screening instruments, the subjective nature of self-reported mood remains a potential source of measurement variability.

Generalizability

The generalizability of these findings is limited by the small and relatively narrow participant pool. The sample may not capture the demographic diversity of broader populations reporting anxiety, depression, or other mental health conditions. Mental health status was self-reported rather than established through clinical assessment. Future studies should include larger, more diverse populations and clinically characterized samples to determine whether the observed behavioral patterns generalize beyond the present cohort.

Comparability

No validated clinical screeners such as the PHQ-9 [4] or GAD-7 [5] were administered alongside Feels.ai data. Mental health status was self-reported and was not independently confirmed through diagnostic interviews or clinical assessment. Information regarding psychiatric comorbidities, symptom severity, treatment status, or suicidal ideation was not collected. Accordingly, the self-reported anxiety and depression categories should not be interpreted as clinically established diagnoses. Furthermore, app-derived behavioral features should not be interpreted as measures of suicide risk or clinical severity.

Tracking Duration

Tracking duration varied across participants, which may limit characterization of longer-term mood patterns. Future studies should use standardized, extended follow-up periods to better evaluate longitudinal mood variability.

Sample Constraints

The small sample size (n=61) limits the precision, stability, and generalizability of the predictive modeling results. This limitation is particularly important given the small number of participants within individual self-reported mental health groups. Although repeated cross-validation was used to reduce dependence on a single train-test split, internal resampling cannot substitute for validation in an independent cohort. The confidence intervals around model performance and a calibration slope below 1 indicate uncertainty in model performance and the potential for overfitting. Accordingly, the classifier should be considered exploratory and hypothesis-generating rather than a validated clinical prediction model. Larger and more diverse samples with external validation are needed before clinical performance or generalizability can be established.

Missing Data and Feature Calculation

Mood skewness and rolling mood volatility could not be calculated for participants with insufficient mood observations. These missing values were median-imputed within cross-validation during model preprocessing. Skewness was noncalculable for 22 of 61 participants (36.1%) and volatility for 27 of 61 (44.3%), with particularly high missingness in the depression group (7/9 and 8/9 participants, respectively). Consequently, the apparent importance of these longitudinal features should be interpreted cautiously, particularly among participants with limited engagement.

Engagement Among Participants Reporting Depression

Participants in the depression group recorded the lowest number of completed days and mood entries. This limited engagement highlights a potential barrier to sustained data collection among participants reporting depression. Future research should explore adaptive strategies to encourage sustained participation, including built-in support features.

## Conclusions

This feasibility study demonstrates the potential of Feels.ai as a platform for longitudinal mood tracking and exploratory classification of self-reported mental health status. The app successfully captured user-generated mood data from which temporal, emotional, and engagement-related behavioral features were derived. Exploratory modeling suggests that these features contain signals associated with self-reported mental health status. However, model performance and calibration were limited. As such, these findings should be interpreted in the context of the small sample size and substantial missingness in some longitudinal features.

While mood-tracking tools are already commercially available, these findings suggest that longitudinal mood data may contain information beyond routine self-monitoring that warrants further investigation in relation to self-reported mental health status. Future studies should determine whether similar behavioral signals are observed in clinically characterized populations and whether they provide additional value alongside validated clinical measures.

## Appendices

Feels.ai Mood Scale

Prompt: How are you feeling today?

Response options: 😀 😌 😐 ☹️ 😞 😭

**Table 3:** Feels.ai Mood Scale. Participants selected the emoji that best represented their current emotional state. Numeric scores and descriptive labels were not displayed within the application. Unicode emoji are shown as equivalent representations of the FlutterFlow icons used in the Feels.ai application. Emoji selections were internally mapped to a 0-10 continuous distress scale for quantitative analysis.

Emoji	Internal distress score (not visible to participants)
😀	0
😌	2
😐	4
☹️	6
😞	8
😭	10

## References

[1] (2026). World Health Organization. Mental disorders. https://www.who.int/news-room/fact-sheets/detail/mental-disorders.

[2] Guntuku SC, Yaden DB, Kern ML (2017). Detecting depression and mental illness on social media: an integrative review. Curr Opin Behav Sci.

[3] Lake J, Turner MS (2017). Urgent need for improved mental health care and a more collaborative model of care. Perm J.

[4] Kroenke K, Spitzer RL, Williams JB (2001). The PHQ-9: validity of a brief depression severity measure. J Gen Intern Med.

[5] Spitzer RL, Kroenke K, Williams JB, Löwe B (2006). A brief measure for assessing generalized anxiety disorder: the GAD-7. Arch Intern Med.

[6] Drake G, Csipke E, Wykes T (2013). Assessing your mood online: acceptability and use of moodscope. Psychol Med.

[7] Nahum M, Van Vleet TM, Sohal VS (2017). Immediate mood scaler: tracking symptoms of depression and anxiety using a novel mobile mood scale. JMIR Mhealth Uhealth.

[8] Pratap A, Neto EC, Snyder P (2020). Indicators of retention in remote digital health studies: a cross-study evaluation of 100,000 participants. NPJ Digit Med.

[9] Bakker D, Kazantzis N, Rickwood D, Rickard N (2016). Mental health smartphone apps: review and evidence-based recommendations for future developments. JMIR Ment Health.

[10] Firth J, Torous J, Nicholas J, Carney R, Pratap A, Rosenbaum S, Sarris J (2017). The efficacy of smartphone-based mental health interventions for depressive symptoms: a meta-analysis of randomized controlled trials. World Psychiatry.

[11] Harrer M, Adam SH, Baumeister H (2019). Internet interventions for mental health in university students: A systematic review and meta-analysis. Int J Methods Psychiatr Res.

[12] Lipschitz JM, Pike CK, Hogan TP, Murphy SA, Burdick KE (2023). The engagement problem: a review of engagement with digital mental health interventions and recommendations for a path forward. Curr Treat Options Psychiatry.

[13] Mohr DC, Riper H, Schueller SM (2018). A solution-focused research approach to achieve an implementable revolution in digital mental health. JAMA Psychiatry.

[14] Planas R, Yuguero O (2021). Technological prescription: evaluation of the effectiveness of mobile applications to improve depression and anxiety. Systematic review. Inform Health Soc Care.

[15] Barrows PD, Thomas SA (2018). Assessment of mood in aphasia following stroke: validation of the Dynamic Visual Analogue Mood Scales (D-VAMS). Clin Rehabil.

[16] Ringwald WR, King G, Vize CE, Wright AG (2025). Passive smartphone sensors for detecting psychopathology. JAMA Netw Open.

[17] Khademian F, Aslani A, Bastani P (2020). The effects of mobile apps on stress, anxiety, and depression: overview of systematic reviews. Int J Technol Assess Health Care.

[18] Wang K, Varma DS, Prosperi M (2018). A systematic review of the effectiveness of mobile apps for monitoring and management of mental health symptoms or disorders. J Psychiatr Res.

[19] Linardon J, Xie Q, Swords C, Torous J, Sun S, Goldberg SB (2025). Methodological quality in randomised clinical trials of mental health apps: systematic review and longitudinal analysis. BMJ Ment Health.

[20] Alyami M, Giri B, Alyami H, Sundram F (2017). Social anxiety apps: a systematic review and assessment of app descriptors across mobile store platforms. Evid Based Ment Health.

[21] Linardon J, Cuijpers P, Carlbring P, Messer M, Fuller-Tyszkiewicz M (2019). The efficacy of app-supported smartphone interventions for mental health problems: a meta-analysis of randomized controlled trials. World Psychiatry.

[22] Wu A, Scult MA, Barnes ED, Betancourt JA, Falk A, Gunning FM (2021). Smartphone apps for depression and anxiety: a systematic review and meta-analysis of techniques to increase engagement. NPJ Digit Med.

[23] Wong DL, Baker CM (1988). Pain in children: comparison of assessment scales. Pediatr Nurs.

[24] Torous J, Lipschitz J, Ng M, Firth J (2020). Dropout rates in clinical trials of smartphone apps for depressive symptoms: a systematic review and meta-analysis. J Affect Disord.

[25] Kelders SM, Kok RN, Ossebaard HC, Van Gemert-Pijnen JE (2012). Persuasive system design does matter: a systematic review of adherence to web-based interventions. J Med Internet Res.

[26] Insel TR (2017). Digital phenotyping: technology for a new science of behavior. JAMA.

[27] Onnela JP, Rauch SL (2016). Harnessing smartphone-based digital phenotyping to enhance behavioral and mental health. Neuropsychopharmacology.

[28] Wang R, Chen F, Chen Z (2014). StudentLife: assessing mental health, academic performance and behavioral trends of college students using smartphones. Proc ACM Int Jt Conf Pervasive Ubiquitous Comput (UbiComp '14).

[29] Xu Z, Gao Y, Wang F, Zhang L, Zhang L, Wang J, Shu J (2025). Depression detection methods based on multimodal fusion of voice and text. Sci Rep.

